# Initial stage of crusted scabies and possible diagnostic characteristics: A case series

**DOI:** 10.1017/S0031182025000113

**Published:** 2025-02

**Authors:** Wanchen Li, Lin Song, Tao Guo, Yaliu Wu, Xiaoli Li, Hongfeng Li, Jianjun Li, Simiao Li

**Affiliations:** 1 Department of Clinical Laboratory, The Second Hospital of Tianjin Medical University, Tianjin, PR China; 2 Department of Clinical Laboratory, Tianjin Academy of Traditional Chinese Medicine Affiliated Hospital, Tianjin, PR China; 3 Department of Dermatology, Tianjin Academy of Traditional Chinese Medicine Affiliated Hospital, Tianjin, PR China

**Keywords:** crusted scabies, diagnosis, *Sarcoptes scabiei*, scabies

## Abstract

Scabies is a neglected tropical disease caused by the ectoparasitic mite, *Sarcoptes scabiei* var. *hominis* (*S. scabiei*). Common scabies, the most prevalent clinical subtype of scabies, is characterized by pruritus, multiple skin lesions and low mite burden. In contrast, crusted scabies, an extremely contagious variant, is characterized by hyperkeratosis and high mite burden, with or without pruritus. Scabies can be diagnosed based on clinical manifestations, with confirmation obtained through microscopic identification of diagnostic features of *S. scabiei*. However, owing to the diversity and non-specific nature of its clinical manifestations and insufficient knowledge regarding early-stage clinical manifestations, the diagnosis of crusted scabies continues to be delayed. Herein, we present three cases of scabies with varying degrees of crusting and mite burden. Three patients with physical and microscopic results suggesting scabies were selected for this study. Case 1 had mild crusting and low mite burden, case 2 had severe crusting and high mite burden and case 3 had mild crusting and high mite burden. In this case report, ‘the initial stage of crusted scabies’ refers to the progression from common to crusted scabies. The discussion regarding the diagnostic characteristics of the initial stage of crusted scabies is expected to aid the early diagnosis of crusted scabies.

## Graphical abstract

**Figure:**
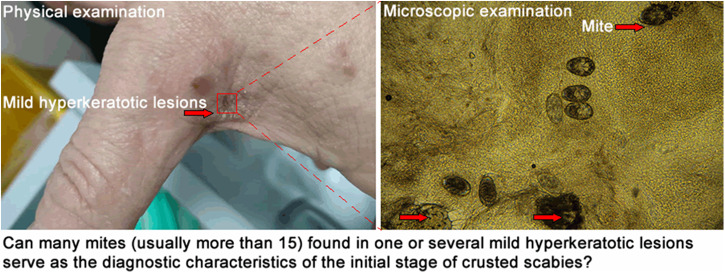

## Introduction

Scabies is a common ectoparasitic dermatosis caused by the mite, *Sarcoptes scabiei* var. *hominis* (*S. scabiei*), which affects approximately 204 million individuals globally (GBD 2015 Disease and Injury Incidence and Prevalence Collaborators, 2016). Although scabies is a public health issue in all countries, this infestation is more common in underprivileged and resource poor settings. The occurrence of scabies in developed countries is often due to institutional outbreaks (Arora et al., 2020).

Scabies can be diagnosed based on clinical manifestations, with confirmation obtained through microscopic identification of diagnostic features of *S. scabiei* (Engelman et al., 2019). Unfortunately, common scabies is often initially misdiagnosed as other dermatoses, such as eczema, psoriasis and atopic dermatitis (Niode et al., 2022). Clinically, these inflammatory dermatoses are frequently treated with corticosteroids (Gavra et al., 2024; Memory et al., 2024). However, corticosteroid use has been considered the most common risk factor for the development of crusted scabies (Bergamin et al., 2024). Owing to a lack of specific clinical signs, the initial stage of crusted scabies is often undetected and misdiagnosed as other hyperkeratotic diseases, such as eczema and psoriasis (Skayem et al., 2023). As scabies is currently recognized as a neglected tropical disease by the World Health Organization, increased awareness and efforts are dedicated to addressing the gap in its diagnosis (Arora et al., 2020). To the best of our knowledge, only a few studies have focused on the diagnostic characteristics of the initial stage of crusted scabies.

Herein, we report three cases of scabies with varying degrees of crusting and mite burden. To date, this is the first case report to explore the diagnostic characteristics of the progressive stage from common to crusted scabies.

## Materials and methods

### Ethics statement

This study was conducted at the Tianjin Academy of Traditional Chinese Medicine Affiliated Hospital in Tianjin, China. The hospital ethics committee approved and monitored the study. Patients or their families provided written informed consent for their participation in this study.

### Physical examination

Physical examination, which involved the identification of the presence/absence of pruritus, and morphology and location of skin lesions, was conducted by a dermatologist with more than 3 years of clinical experience. Data regarding hyperkeratosis, including the depth of crust and degree of skin shedding, were recorded in detail. According to the severity grading scale for crusted scabies described elsewhere (Davis et al., 2013), the degree of crusting was divided into three grades.

1. Mild crusting: <5 mm depth of crust, minimal skin shedding;
2. Moderate crusting: 5–10 mm depth of crust, moderate skin shedding; and
3. Severe crusting: >10 mm depth of crust, profuse skin shedding.

### Microscopic examination

Microscopic examination was performed by operators with more than three years of clinical experience as described elsewhere (Li et al., 2023). First, several suspected sites were determined based on skin lesion morphologies at finger web-spaces, hands, the volar surfaces of the wrists, axillae, buttocks, the areola in women, the toe seams in infants or young children, and genitalia in men. After disinfection with 75% alcohol, skin scrapings were collected from the suspected sites using a sterile blade (#15), carefully placed on a standard glass microscope slide, mixed with one–two drops of 15% potassium hydroxide, and covered with a coverslip. After several minutes, the slide was scanned under a microscope (Olympus CX33; Tokyo, Japan) at 100× magnification to search for the diagnostic features of *S. scabiei*. All fields under the microscope were recorded as video files using a JEDA SmartVF650DO camera (Jiangsu, China) equipped with an automated imaging system. Representative images presented in this case report were selected using Adobe Premiere Pro CC 2020 (Adobe Systems Software Ireland Limited, Dublin, Ireland) and edited using Adobe Fireworks CS4 (Adobe Systems Software Ireland Limited, Dublin, Ireland).

Mite count is the sum of complete mites and gnathosoma-type mite fragments (incomplete mites with gnathosoma) (Li et al., 2023). Mite burden was estimated and divided into three grades.

1. Low mite burden: <5 mites;
2. Moderate mite burden: 5–15 mites; and
3. High mite burden: >15 mites.

### Treatment

The anti-scabies treatment was 10% sulphur ointment (applied everywhere below the neck, especially on wrinkles). Treatment was applied daily for 3 days; patients were not allowed to bathe during the treatment period. After 3 days, patients took a bath and donned new clothes. Treatment was discontinued for 3 days, and a follow-up course of treatment was performed, if necessary. Systemic antibiotics and pruritus relieving agent (dexamethasone acetate cream) were used, if necessary. All treatments were performed at home under the supervision of the patient’s family.

## Participants and data collection

Three outpatients who underwent physical and microscopic examination were selected:

1. One with mild crusting and low mite burden;
2. One with severe crusting and high mite burden; and
3. One with mild crusting and high mite burden.

Demographic data, such as age, sex, comorbidities, and medical history, were collected. Clinical data included the results of physical and microscopic examination and the treatment process.

## Case presentation

### Case 1

**Figure 1.**
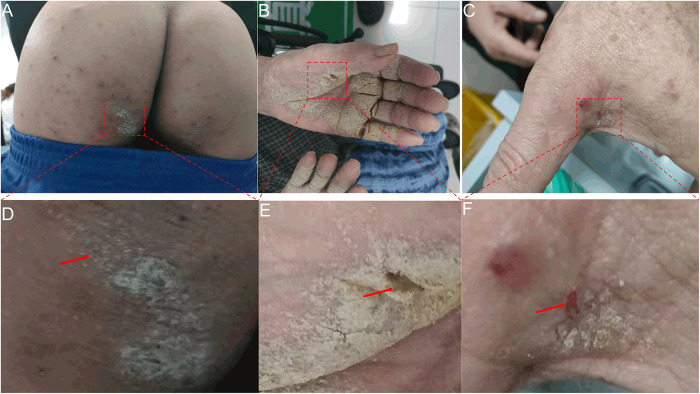
Representative skin lesions and sampling sites. A: mild crusting on the left buttock of the patient in case 1; B: severe crusting on the hands of the patient in case 2; C: mild crusting between the thumb and index finger of the patient in case 3. D, E and F are partially enlarged pictures (5×). Red arrow: sampling sites.

**Figure 2.**
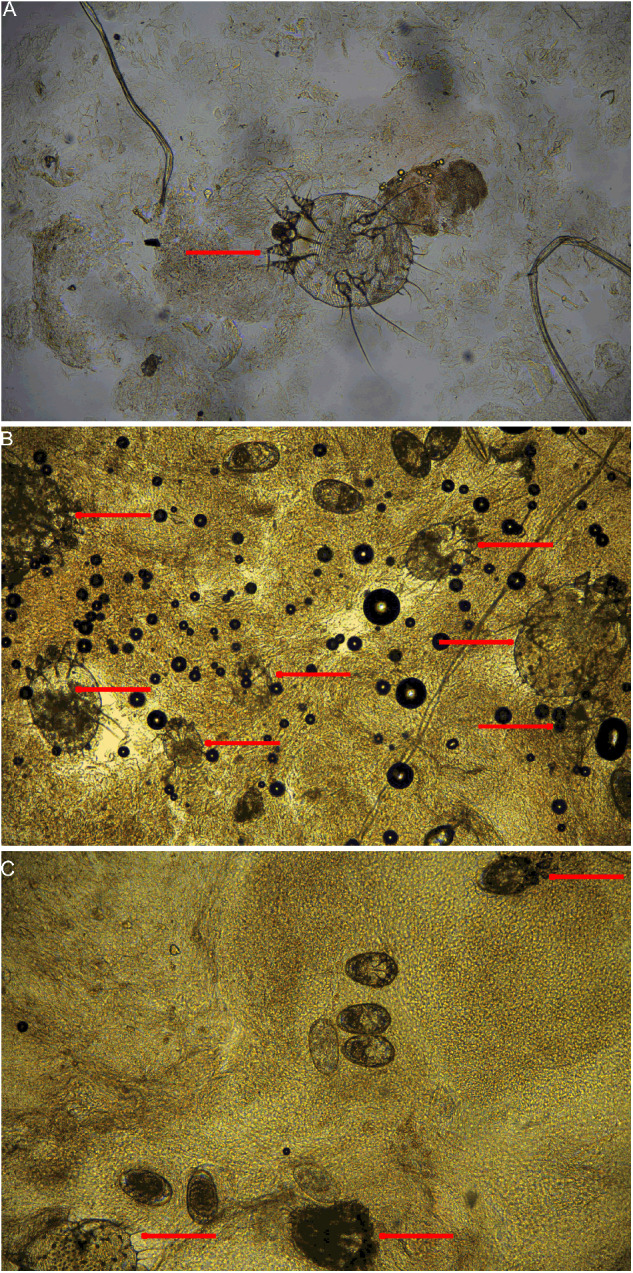
Representative fields of mites identified in the microscopic examination of case 1 (A), case 2 (B) and case 3 (C). Red arrow: mites (magnification: 100).

In early December 2023, a 14-year-old male with papules and pruritus visited the outpatient department of another hospital and was diagnosed with eczema. Topical corticosteroid was used twice but was ineffective. Consequently, the patient opted to discontinue treatment application. On December 27, the patient visited the outpatient department of our hospital for treatment. Physical examination revealed scattered red papules on the trunk, scales and blisters in the finger web-spaces, and mild crusting on the buttocks (Figure 1A). Microscopic examination revealed one mite in the skin scrapings collected from the hyperkeratotic lesions on the left buttock (Figure 2A). After two courses of anti-scabies treatment, the infestation was cured.

### Case 2

In July 2023, a 92-year-old female nursing home patient had a persistent and worsening rash and pruritus that led to months of discomfort. The patient has a history of cerebral infarction and cannot move independently. The patient visited the outpatient department of another hospital two months prior to visiting our hospital and was diagnosed with eczema. Dexamethasone acetate cream was applied 2–3 times daily until her visit to our hospital. The pruritus was relieved at the beginning of treatment; however, her symptoms became increasingly severe. Physical examination revealed scattered papules on the trunk and limbs, reddish brown nodules on the buttocks, and severe crusting on the hands (Figure 1B). Microscopic examination revealed 49 mites in the skin scrapings shed from the hyperkeratotic lesions on the left hand (Figure 2B). Dexamethasone acetate cream was discontinued, and anti-scabies treatment was applied. After three courses of treatment, the hyperkeratotic lesions disappeared. Owing to the skin irritation induced by sulphur, pruritus appeared, and dexamethasone acetate cream was applied. After approximately one month, the patient developed a rash and pruritus. Microscopic examination confirmed the detection of scabies (data not shown). After four courses of anti-scabies treatment, symptoms disappeared. The patient’s last follow-up visit was on January 1, 2024. No skin lesions were found, and the microscopic result was negative for scabies.

### Case 3

In late July 2023, a 69-year-old male was admitted to a hospital due to myocardial infarction. Following hospitalization for half a month, the patient was transferred to another hospital due to ongoing physical discomfort. After hospitalization for another half a month, the patient was discharged. According to the patient’s family, no skin lesions or pruritus occurred during this period; however, papules and pruritus began to appear in early September. The patient visited the outpatient department of another hospital and was diagnosed with eczema. Dexamethasone acetate cream was applied 2–3 times daily until his visit to our hospital; however, his symptoms kept worsening. On October 26, 2023, the patient visited the outpatient department of our hospital. Physical examination revealed many brown pigmentation spots on the trunk and limbs, red nodules on the scrotum and buttocks, and mild crusting in the finger web-spaces (Figure 1C). Microscopic examination revealed 21 mites in the skin scrapings collected from the hyperkeratotic lesions between the thumb and index finger (Figure 2C). Dexamethasone acetate cream was discontinued, and anti-scabies treatment was applied (two courses of treatment). After two weeks, the patient was given another two courses of treatment. The last follow-up visit occurred approximately a month later. No skin lesions were found, and the microscopic result was negative for scabies.

## Discussion

To our knowledge, this is the first published description of the initial stage of crusted scabies. As shown in case 3, the initial stage of crusted scabies refers to the progressive stage from common to crusted scabies, which appears similar to common scabies but involves many mites (usually more than 15) in single or several mild hyperkeratotic lesions.

Generally, scabies can be divided into different clinical subtypes. As presented in case 1, common scabies is characterized by pruritus with multiple skin lesions, including excoriated papules, eczematous or lichenified plaques, and nodules. In contrast, crusted scabies is a rare and severe variant that is characterized by hyperkeratosis with or without pruritus (Motswaledi, 2021), as presented in case 2. It has been reported that untreated crusted scabies carries a high risk of mortality (Currie et al., 1997; Hasan et al., 2020). However, these manifestations are non-specific and resemble other skin conditions, including eczema, psoriasis, atopic dermatitis, diaper rash, insect bites and poison ivy (Arlian and Morgan, 2017; Niode et al., 2022). Visualization methods, including microscopic examination, videodermoscopy, reflectance confocal microscopy and handheld dermoscopy, are required to confirm the diagnosis of scabies (Engelman et al., 2020). Unfortunately, these methods are insensitive – a negative result cannot exclude diagnosis, leading to frequent misdiagnosis (Engelman et al., 2020). According to a single-centre retrospective study conducted in the United States, 45.3% of patients with scabies were initially misdiagnosed (Anderson and Strowd, 2017).

Misdiagnosis is associated with two serious consequences: incorrect treatments being prescribed to patients (mainly topical corticosteroid use) and more infections being transmitted to the contacts of affected patients (Skayem et al., 2023). The disease pathology of crusted scabies involves both non-protective allergic Th2 and pro-inflammatory IL-17 responses (Bhat et al., 2017). A normal cell-mediated immune response is thought to limit the proliferation of mites and prevent over-infestation and the subsequent development of crusted scabies (Bergamin et al., 2024). Corticosteroid-induced impairments to inflammatory and cellular immune responses facilitate mite proliferation and subsequent skin pathology (Bergamin et al., 2024). Therefore, corticosteroid use has been considered the most common risk factor for the development of crusted scabies (Bergamin et al., 2024).

Crusted scabies usually occurs in individuals with defective T-cell immunity (HIV, leukaemia and lymphoma), reduced cutaneous sensation (leprosy and neurological disorders), and reduced ability to mechanically debride the mites (critical illness, Down’s syndrome and senile dementia) (Arora et al., 2020). In addition, crusted scabies is commonly misdiagnosed in transplant recipients (Wang et al., 2019). Notably, individuals without identifiable immunodeficiency or risk factors can also develop crusted scabies. According to some investigators, individuals may have a genetic predisposition to this condition (Bhat et al., 2017). However, other investigators believe that the immune defect in individuals with crusted scabies is multifactorial (Walton, 2010).

As mentioned above, the mite burden differs between common scabies and crusted scabies. Most individuals with common scabies have a low mite burden, with an average of <20 (5–15) mites on the entire body (Davis et al., 2013; Thomas et al., 2020). In contrast, individuals with crusted scabies have a high mite burden, with hundreds, thousands, or even millions of mites per patient (Bernigaud et al., 2020). Previously, approximately 4,700 mites were obtained in 1 g of skin scrapings shed from a patient with hyperkeratosis (Walton et al., 1999).

Under normal circumstances, the outer skin barrier’s cornified layers are continuously shed by desquamation, a unique programmed cell death called keratinization (Eckhart et al., 2013). Following infection with *S. scabiei*, the cuticle of the host skin is destroyed. To repair the damaged skin barrier, energy is needed, which is released by mitochondrial metabolism. High mite burden leads to mitochondrial depletion and abnormalities. Cytochrome C is released after abnormal swelling of the mitochondria, which leads to caspase-mediated cell death (Lee et al., 2021), inducing excessive desquamation and eventually crust formation. This view was proposed and confirmed using a rabbit model of crusted scabies (Guan et al., 2023).

A severity grading scale for crusted scabies was developed based on clinical assessment of four key areas: distribution and extent of crusting, depth of crusting, degree of skin cracking and pyoderma, and number of previous episodes (Davis et al., 2013). Although patients with the advanced stage of the disease are more likely to be diagnosed, the diagnosis of crusted scabies is still delayed. According to Yélamos et al., up to several months of delay can occur, with a median of 7 months and a maximum of up to 16 months (Yélamos et al., 2016). Such delay may be due to the lack of specific clinical manifestations of crusted scabies and insufficient knowledge of their clinical presentation (Skayem et al., 2023). Consequently, clarifying the diagnostic characteristics of the initial stage of crusted scabies may contribute to solving the problem of the delayed diagnosis of this disease. We believe that the identification of multiple mites in single or several mild hyperkeratotic lesions can serve as an objective diagnostic characteristic of the initial stage of crusted scabies.

To date, no sensitive method has been established for identifying mites or estimating mite burden. For microscopic examination, the key point is to obtain high quality skin scrapings. Several useful skills are shared. First, the operator should be skilled and familiar with multiple skin lesion morphologies of common scabies, crusted scabies and other skin diseases that are difficult to decipher from scabies. Second, the feeling of scraping off materials from individuals with scabies can be slightly different from that from individuals with eczema or psoriasis. The connection between some lesions and surrounding tissues in individuals with scabies seems to be looser than that in individuals with eczema or psoriasis, enabling easier retrieval of the scrapings. This looser connection may be due to the damage induced by mites occurring via two processes: physical (digging burrows) and chemical (secreting saliva to dissolve the stratum corneum) (Arlian and Morgan, 2017). However, practice is required to make this distinction. Moreover, this looser connection may not always occur. Third, if the initial stage of crusted scabies is suspected according to clinical manifestations, but ≤15 mites are found, the diagnosis should be considered carefully and, where possible, more skin scrapings should be obtained.

This case report has some limitations. First, whether mites can be transmitted from individuals at the initial stage of crusted scabies to their contacts via fomites needs further study. Generally, mites are transmitted through skin-to-skin or sexual contact (Bogino et al., 2023). Furthermore, transmission of mites via fomites does not play a major role in common scabies but is important in crusted scabies owing to the extremely high mite burden (Arora et al., 2020; Engelman et al., 2020). Previously, a rabbit was found to be infected within 31 min of contact with live human scabies mites obtained from the bed linen of patients heavily infected with scabies (Arlian et al., 1984).

Second, whether additional treatments should be administered to individuals at the initial stage of crusted scabies also needs further study. Common scabies is traditionally treated with oral medications and/or topical scabicides, including 5% permethrin, 10–20% benzyl benzoate, 2–10% precipitated sulphur, 10% crotamiton, 0.5% malathion and 1% lindane (Thomas et al., 2020). As these medications are not ovicidal, a second dose is required to kill newly hatched mites. Ivermectin is a common oral medication but is not approved for treating scabies in China. In our hospital, 5–10% precipitated sulphur is the only medication that can be used to treat scabies. For crusted scabies, additional treatments (topical keratolytics, systemic antibiotics and topical corticosteroids) can be applied where clinically necessary (Davis et al., 2013; Skayem et al., 2023). It should be noted that environmental decontamination and patient isolation also play vital roles in the treatment of crusted scabies (Salavastru et al., 2017; Palaniappan et al., 2021). In addition, in drug-resistant or severe cases, the use of multi-dose ivermectin protocols in conjunction with topical scabicides is encouraged (Niode et al., 2022).

## Conclusion

Further investigations involving a larger number of cases are necessary to gain additional evidence to support the diagnostic characteristics of the initial stage of crusted scabies and elucidate the mechanisms underlying the clinical manifestations. Importantly, the discussion regarding the diagnostic characteristics of the initial stage of crusted scabies may be beneficial to the early diagnosis of crusted scabies and understand the progression from common to crusted scabies.

## Data Availability

The complete datasets are available upon reasonable request.
